# Supplementary material for: Ethical challenges in palliative sedation of adults: protocol for a systematic review of current clinical practice guidelines
Source: BMJ Open. 2022 Jul 1;12(7):e059189. doi: 10.1136/bmjopen-2021-059189 (PMC9252196; doi:10.1136/bmjopen-2021-059189)
Supplement: Supplementary data [file bmjopen-2021-059189supp001.pdf]

BMJ Publishing Group Limited (BMJ) disclaims all liability and responsibility arising from any reliance placed on this supplemental material which has been supplied by the author(s)

Draft Embase strategy, 29.06.2021

https://www.embase.com/#search

((('sedation'/exp OR sedation:ab,ti,kw) AND ('palliative therapy'/exp OR 'terminal care'/exp OR 'palliative nursing'/exp)) OR (((palliative OR terminal) NEAR/6 sedation) OR (continuous NEAR/3 sedation)):ab,ti,kw) AND ('practice guideline'/de OR 'consensus development'/de OR (guideline* OR recommendation* OR statement* OR "position paper" OR consensus):ti,kw) AND [2000-2021]/py NOT ('juvenile'/exp NOT 'adult'/exp)
